# Chlortetracycline Concentration Impact on *Salmonella* Typhimurium Sustainability in the Presence of Porcine Gastrointestinal Tract Bacteria Maintained in Continuous Culture

**DOI:** 10.3390/pathogens12121454

**Published:** 2023-12-15

**Authors:** Dana K. Dittoe, Robin C. Anderson, Toni L. Poole, Tawni L. Crippen, Roger B. Harvey, Steven C. Ricke

**Affiliations:** 1 Department of Animal Science, University of Wyoming, Laramie, WY 82071, USA; ddittoe@uwyo.edu; 2 Food and Feed Safety Research Unit, United States Department of Agriculture, Agricultural Research Service, Southern Plains Agricultural Research Center, College Station, TX 77845, USA; robin.anderson@usda.gov (R.C.A.); toni.poole@usda.gov (T.L.P.); tc.crippen@usda.gov (T.L.C.); roger.harvey@usda.gov (R.B.H.); 3 Meat Science and Animal Biologics Discovery Program, Department of Animal and Dairy Sciences, University of Wisconsin, Madison, WI 53706, USA

**Keywords:** *Salmonella* Typhimurium, chlortetracycline, porcine, continuous culture

## Abstract

Concern exists that the continued use of antibiotics in animal feeds may lead to an increased prevalence of resistant bacteria within the host animal’s gastrointestinal tract. To evaluate the effect of chlortetracycline on the persistence of *Salmonella enterica* serotype Typhimurium within a diverse population of porcine cecal bacteria, we cultured a mixed population of cecal bacteria without or with added chlortetracycline. When grown at a 24 h vessel turnover rate, chlortetracycline-susceptible *S.* Typhimurium exhibited more than 2.5 times faster (*p* < 0.05) disappearance rates than theoretically expected (0.301 log_10_ colony-forming unit/mL per day) but did not differ whether treated or not with 55 mg of chlortetracycline/L. Chlortetracycline-resistant *S.* Typhimurium was not recovered from any of these cultures. When the mixed cultures were inoculated with a chlortetracycline-resistant *S.* Typhimurium, rates of disappearance were nearly two times slower (*p* < 0.05) than those observed earlier with chlortetracycline-susceptible *S.* Typhimurium, and cultures persisted at >2 log_10_ colony-forming units/mL for up to 14 days of treatment with 110 mg of chlortetracycline/L. Under the conditions of this study, chlortetracycline-resistant *S.* Typhimurium was competitively enabled to persist longer within the mixed populations of porcine gut bacteria than chlortetracycline-susceptible *S*. Typhimurium, regardless of the presence or absence of added chlortetracycline.

## 1. Introduction

Historically, antimicrobial feed additives have been employed in the animal industry to improve growth rate and feed utilization and decrease mortalities from bacterial infections [1]. Broom has summarized some of the beneficial mechanisms that have been proposed for feeding subinhibitory concentrations of antibiotics to farm animals, including decreasing total microbial density in the gastrointestinal tract (GIT), reducing sub-clinical infections, limiting the generation of potentially toxic bacterial metabolites, improving absorption of nutrients, and limiting intestinal inflammatory cell deleterious impact [2]. However, the widespread use of antibiotics as feed additives, especially when administered at prophylactic doses, has raised concerns that an antibiotic-resistant bacterial community may be retained in the animal and, ultimately, in the human environment [3,4]. To make this an even more critical issue, pathogens such as *Salmonella* possess numerous mobile genetic elements that collectively comprise a resistance mobilome that is easily transferable among *Salmonella* isolates and related species [5]. This is of particular concern with food animals such as swine, which can be a reservoir for antibiotic-resistant *Salmonella,* and the resulting pork products as a source of human salmonellosis [6,7,8].

*Salmonella* subclinical GIT colonization and infection occur in swine, with numerous isolates exhibiting multiple drug resistance [6,9,10]. Among the antibiotics administered, tetracyclines have been some of the more common ones in use for therapeutic purposes in the swine industry in the U.S. [9]. Tetracyclines exhibit antimicrobial activity via inhibition of protein synthesis in bacterial cells by attachment to the 30S ribosomal subunit, thus preventing aminoacyl-tRNA binding to the mRNA-ribosome complex [11,12]. Derivatives such as chlortetracycline have been extensively used for growth promotion, but banning the use of tetracyclines as growth promoters in the European Union has not diminished the occurrence of tetracycline resistance in *Salmonella* from swine [11]. This is particularly true for *S*. Typhimurium, which appears to possess transferable mobile genetic elements that may support the ready acquisition of tetracycline resistance [11].

While antibiotics are administered orally or via injection, they are also included as a feed additive [9,13]. However, Barton points out that the problem with feed administration is ensuring that the pig consumes the appropriate dose of the antibiotic, and if this does not occur, it can lead to increased antibiotic transmission risk [9]. It has been shown that commensal GIT bacteria can serve as a preventative barrier to the colonization of antibiotic-resistant pathogens [14]. The purpose of the study presented here was to determine the persistence of antibiotic-sensitive and resistant *Salmonella* in mixed porcine GIT bacterial continuous flow cultures exposed to either one-fold or two-fold levels of chlorotetracycline. This model system has been used previously to study the persistence of *Salmonella* Typhimurium in mixed populations of avian and porcine GIT bacteria [15,16,17].

## 2. Materials and Methods

### 2.1. Bacterial Strains, Isolation, and Cultivation

Chlortetracycline-sensitive and -resistant *Salmonella* Typhimurium were swine isolates provided to our laboratory in 1995 and 1997, respectively, by the USDA National Veterinary Services Laboratory (Ames, IA, USA). The chlortetracycline-sensitive *S.* Typhimurium had been isolated from lung tissue. Additional background information on the chlortetracycline-resistant *S.* Typhimurium is unavailable to us. Both strains were grown overnight at 37 °C in Bacto^TM^ Typtic Soy Broth (TSB) (Becton Dickinson and Company, Sparks, MD, USA) prior to inoculation into the continuous flow cultures. All antibiotics were purchased from Sigma (St. Louis, MO, USA) and were prepared according to the manufacturer’s instructions. Chlortetracycline was used at 55 or 110 mg/L, which corresponded to industry usage levels of 50 or 100 g/ton, respectively [18]. Samples collected from mixed cultures were serially diluted (10-fold) in sterile phosphate-buffered saline (PBS, pH. 7.0) followed by plating on selective Brilliant Green Agar (BGA) (Oxoid, Unipath Ltd., Basinstoke, Hampshire, UK) supplemented without or with 55 mg/L chlortetracycline and incubated at 37 °C overnight for enumeration of chlortetracycline-susceptible or -resistant *S*. Typhimurium. Each dilution was plated in triplicate, and all values represent an average.

### 2.2. Continuous Flow Cultures

In order to determine the best method for testing the competitiveness of *Salmonella* within the mixed cecal population, a continuous flow apparatus was inoculated with a mixed population of porcine cecal bacteria as previously described [15,19] or similarly with pure *Salmonella* cultures. In an early report [20], the continuous flow culture was found to contain, at a minimum, the following bacteria: *Enterococcus faecalis*, *Streptococcus bovis*, *Clostridium clostridiforme*, *C. symbiosurn*, *C. ramosum*, *Bacterioides fragilis*, *B. distasonis*, *B. vulgatus*, *B. uniformis*, and *B. caccae*. Moreover, the culture notably did not contain indigenous *Escherichia coli*. This porcine-derived continuous flow culture has been used in previous studies to demonstrate the population’s competitive exclusion ability against experimentally inoculated *Salmonella* and *Escherichia coli* as well as to investigate the effects of vancomycin treatments on the persistence of an experimentally inoculated vancomycin-resistant *Enterococcus faecium* [15,20,21]. In the present study, 1150 mL BioFlo chemostat vessels (New Brunswick Scientific Company, Edison, NJ, USA) containing 1050 mL of Viande Levure (VL) [22] broth were inoculated with mixed cecal bacteria [15] or with *S.* Typhimurium and incubated anaerobically at 39 °C while agitated at 100 rpm. The culture medium was prepared and maintained anaerobically under a continuous stream of nitrogen gas. Unless otherwise stated, the dilution rate of the continuous flow culture was 0.0416 per hour, equivalent to a 0.80 mL/min flow rate and a 24 h vessel turnover time. The steady state of continuous flow-mixed cecal bacterial cultures occurred after 14 vessel turnovers, at which time 100 mL of an overnight-grown batch culture of *S.* Typhimurium was added. Pure continuous flow cultures of *S.* Typhimurium reached a steady state after 3 vessel turnovers and were challenged by successively adding 2.5 mL of the mixed porcine culture every 2 h for 8 h. Small volumes (less than 10 mL) were then removed from the continuous flow cultures at intervals and enumerated for *S.* Typhimurium as previously described. For cultures treated with chlortetracycline, treatments were administered immediately after a challenge with additions to both the medium reservoir and chemostat vessel.

### 2.3. Statistics

Results from the initial non-replicated study assessing two different procedures for establishing *S.* Typhimurium in continuous flow cultures are presented descriptively. Due to the die-off of the chlortetracycline-susceptible *Salmonella* in subsequent cultures, only the first 9 days were included in all analyses. The colony-forming units (CFU)/mL of *Salmonella* Typhimurium over the first 9 days were log_10_-transformed and analyzed in JMP Pro 16 (SAS, Cary, NC, USA). The continuous response of *Salmonella* (log_10_ CFU/mL) was analyzed as an indicator-variable regression (ANCOVA), with the day (0 to 9) being considered the continuous variable and treatment as the nominal variable (resistant control, resistant to 2× chlortetracycline, susceptible control, susceptible to 1× chlortetracycline, and theoretical). The main effect and interaction of treatment and time were explored, with the slopes of the treatments across time being compared with the overall average using ANCOVA. Significance was determined at *p* ≤ 0.05.

## 3. Results

A descriptive assessment of the two different established procedures revealed that initial rates of *S*. Typhimurium washout were similar regardless of whether the continuous flow cultures were first established with a pure culture of *S.* Typhimurium and then inoculated with mixed cecal bacteria or vice versa. For both procedures, *S.* Typhimurium decreased from 10^8^ CFU/mL upon initiation of competition to about 10^3^ CFU/mL by day 7 of culture. The continuous cultures, regardless of establishment procedure, achieved rates of decrease in *S.* Typhimurium ranging from 0.411 to 0.544 log_10_ CFU/mL per day over the first nine days of culture, and thereafter, the *Salmonella* populations decreased more gradually in a similar fashion to about 10 CFU/mL by 30 days of culture. Subsequent experiments were conducted by adding *Salmonella* to established cultures of mixed cecal bacteria following a 14-day adaption period (the equivalent of 14 vessel turnovers) that allowed for the mixed populations of porcine cecal bacteria to reach a steady state.

**Figure 1:**
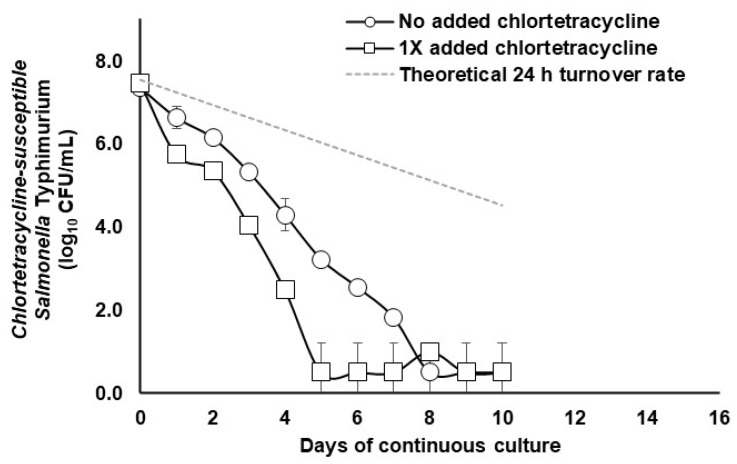
Clearance of chlortetracycline-susceptible *S*. Typhimurium (log_10_ CFU/mL) from mixed populations of porcine gut bacteria during continuous flow culture at a 24 h vessel turnover rate in the absence (open circles) or presence (open squares) of 50 g/ton added chlortetracycline (0.0055%). The dashed line represents the theoretical clearance rate of static *S.* Typhimurium cells neither propagating nor dying during cultivation. Error bars indicate the standard deviation from *n* = 2 cultures/treatment.

**Figure 2:**
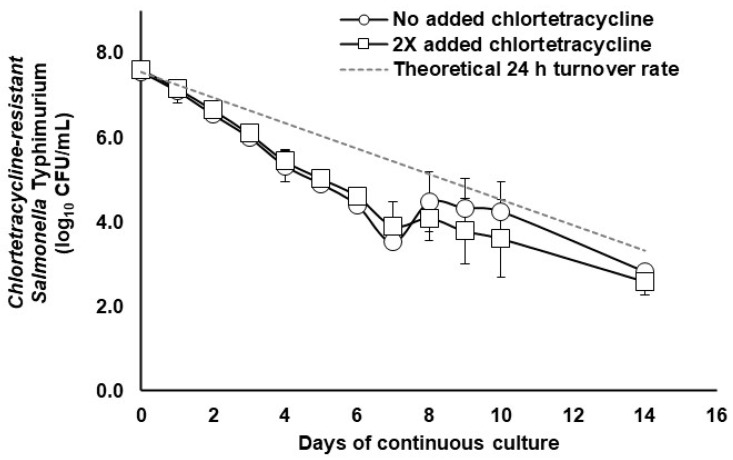
Clearance of chlortetracycline-resistant (*CTC-resistant*) *S*. Typhimurium (log_10_ CFU/mL) from mixed populations of porcine gut bacteria during continuous flow culture at a 24 h vessel turnover rate in the absence (open circles) or presence (open squares) of 100 g/ton added chlortetracycline (0.011%). The dashed line represents the theoretical clearance rate of static *S.* Typhimurium cells neither propagating nor dying during cultivation. Error bars for cultures treated with 100 g/ton added chlortetracycline indicate the standard deviation from *n* = 2 cultures/treatment. Untreated control values are from a single continuous flow culture.

In the absence or presence of 55 mg/L chlortetracycline, chlortetracycline-susceptible *S.* Typhimurium was reduced to undetectable levels in the continuous flow cultures of mixed cecal populations within 8 days (Figure 1). However, from day 2 to day 7, in the presence of chlortetracycline, the clearance rate of the chlortetracycline-susceptible *S.* Typhimurium strain increased, resulting in *S.* Typhimurium achieving washout 3 days earlier than the non-chlortetracycline-treated control cultures. Chlortetracycline-resistant *S.* Typhimurium was not detected throughout this ten-day study, likely because there were too few *S.* Typhimurium cells to support the likelihood of resistance development. With respect to continuous flow cultures of the mixed population of cecal microbes cultured with the chlortetracycline-resistant *S.* Typhimurium, the chlortetracycline-resistant *S.* Typhimurium populations decreased from the continuous flow culture at almost the same rate, whether in the presence or absence of 110 mg/mL added chlortetracycline (Figure 2).

When comparing the slopes of the different *Salmonella* (log_10_ CFU/mL) cultures, the resistant control was resistant to 2× chlortetracycline, the susceptible control was susceptible to 1× chlortetracycline, and theoretically, there was an interaction between treatment (*Salmonella* cultures) and day (*p* < 0.0001; Table 1). This indicates that there was a significant difference between the slopes of the treatments, which are expressed as clearance rates for the treatments. To further delineate these differences, the clearance rates of the different *Salmonella* cultures were compared to the average clearance rate (0.608 log_10_ CFU/mL per day). The resistant *Salmonella* cultures (resistant control, resistant to 2× chlortetracycline) and the theoretical treatment had slower clearance rates (0.410, 0.451, and 0.301 log_10_ CFU/mL per day) than the average (0.608 log_10_ CFU/g per day; *p* < 0.05). The clearance rates of the susceptible *Salmonella* cultures (susceptible control, susceptible to 1× chlortetracycline) were faster (0.821 and 0.805 log_10_ CFU/mL per day) than the average (0.608 log_10_ CFU/mL per day; *p* < 0.05).

## 4. Discussion

The development of antibiotic-resistant bacteria is of major concern worldwide due to the potential development of resistant human pathogens for which no current method of treatment is available. The treatment of bacterial pathogens with antibiotics has led to concerns over the potential development of antibiotic-resistant pathogens in food animals that can, in turn, serve as potential reservoirs for humans [3,4]. For pathogens such as *Salmonella*, it is this potential reservoir of antibiotic resistance that can lead to transmission by mobile genetic elements of genes associated with antibiotic resistance and widespread dissemination [5]. There is also the risk of the transfer of drug-resistant bacteria from food animals such as swine to humans, as is associated with outbreaks of *S.* Typhimurium [6,9,10,23].

The development and transfer of resistant genes amongst bacteria in vitro using forced filter mating and liquid mating can measure the frequency of genetic exchange; however, in vivo, a transfer is difficult to determine, especially in a mixed complex bacterial population when optimum parameters for transfer are not present [24]. In a stable chemostat containing a mixed bacterial population from the pig GIT, *S.* Typhimurium is unable to persist for more than 30 days [15,17,20]. Moreover, the addition of a small volume of a mixed bacterial population to a pure continuous flow culture of *S.* Typhimurium may effectively be able to outgrow and thereby exclude the pathogen. The mechanism of this exclusion is unclear, but it is possible that restricted nutrient availability, bacteriocin production, or the production of volatile fatty acids, among other unidentified factors, may inhibit the growth of *S.* Typhimurium [14]. The persistence of *S.* Typhimurium for 30 days in the chemostat is similar to the length of time, 21 days, seen in cecal cannulated pigs artificially infected with *S.* Typhimurium [25]. In the chemostat model presented here, the addition of *S.* Typhimurium represents a single exposure to the pathogen, whereas, in the natural environment, the animal could be repeatedly exposed to the pathogen, resulting in a persistent low-level infection.

The addition of antibiotics not only affects the pathogen but also affects susceptible bacteria of the normal animal microbiota. For instance, the microbial population within this porcine-derived continuous flow culture was able to exclude an experimentally inoculated vancomycin-resistant *E. faecium* by 7 days of culture, but following treatment with up to 0.1 µg of vancomycin/mL, the vancomycin-resistant *E. faecium* was able to persist for up to 24 days [21]. Evidence from an additional study indicated that tylosin treatment (25 or 100 µg/mL) enriched numbers of indigenous tylosin-insensitive anaerobes within this porcine-derived continuous flow culture more readily than within a population of cecal microorganisms derived from a feral pig and cultured under similar continuous flow culture conditions [26]. Moreover, the tylosin-insensitive anaerobes persisted at higher concentrations in the continuous flow cultures even after cessation of tylosin treatment, although at a lower level in the cultures derived from the feral pig than the more often studied culture derived from a commercially reared pig [26]. In the present study, the addition of chlortetracycline at a level commonly used as a growth promoter (50 g/ton) caused a rapid decrease in *S.* Typhimurium over the first 5 days as compared to the control culture, but in both treated and untreated cultures, *S.* Typhimurium reached undetectable levels by day 8. This could suggest that without antibiotic treatment, a single high-dose exposure to *S.* Typhimurium can be effectively eliminated by the normal microbiota. Under natural conditions, animals chronically infected with *S.* Typhimurium may represent animals that are continuously exposed to contaminated feed or water or animals whose normal microbiota has been disrupted through disease or exposure to other antibiotics.

Under conditions of antibiotic selection, it is speculated that antibiotic-resistant bacteria could have a selective advantage over the normal susceptible microbiota. A chlortetracycline-resistant *S.* Typhimurium under antibiotic selection was reduced to a similar level as the untreated control after a period of 14 days. The Feed Additive Compendium recommends that the feeding of chlortetracycline should not exceed 14 days, so the experiment was terminated at that time [27]. The data suggest that the acquisition of antibiotic resistance may have a secondary effect that enables the bacterium to persist for a longer time period by disrupting the normal microbiota, despite the selective pressure of the antibiotic. This could be particularly problematic if feed administration is compromised and the pig consumes the incorrect dose of the antibiotic, potentially leading to increased antibiotic transmission risk [9].

## Figures and Tables

**Table 1:** Observed clearance rates and recovery of chlortetracycline-susceptible and -resistant *S.* Typhimurium during continuous culture with mixed populations of porcine cecal bacteria supplemented without or without 55 or 110 mg/L (the equivalent of 50 or 100 g/ton) of chlortetracycline.

	Chlortetracycline-Susceptible *Salmonella* Typhimurium		Chlortetracycline-Resistant *Salmonella* Typhimurium		
	Without Added Chlortetracycline	With 55 mg/L of Chlortetracycline	Without Added Chlortetracycline	With 110 mg/L of Chlortetracycline	SEM
Observed clearance rate (log_10_ CFU/mL per day) ^1^	0.821 ^a^	0.805 ^a^	0.409 ^b^	0.451 ^b^	0.069
Chlortetracycline-resistant *S.* Typhimurium recovered after 9 days continuous culture (log_10_ CFU/mL) ^2^	None recovered	None recovered	4.32	3.04	NA

^1^ The continuous flow cultures were operated at a 24 h fluid volume turnover rate, which theoretically achieves a clearance rate of static (neither propagating or dying) *S.* Typhimurium populations of 0.301 log_10_ CFU *S.* Typhimurium/mL per day. Observed clearance rates were determined as the slope of the log_10_ CFU/mL after 9 days of continuous culture. ^2^ Chlortetracycline-resistant *Salmonella* Typhimurium were recovered via plating of serial 10-fold dilutions to Brilliant Green agar supplemented with 110 mg/L. ^a,b^ The continuous clearance rate response of *Salmonella* (log_10_ CFU/mL) was analyzed as an indicator-variable regression (ANCOVA), with day (0 to 9) being considered the continuous variable and treatment as the nominal variable (resistant control, resistant to 110 mg/L of chlortetracycline treatment; susceptible control, susceptible to 55 mg/L of chlortetracycline treatment; and theoretical). Means with unlike-letter superscripts differ at *p* < 0.05 based on a completely randomized analysis of variance. The theoretical clearance rate of 0.301 log_10_ CFU *S.* Typhimurium/mL per day differed significantly from the clearance rates of the chlortetracycline-susceptible *S.* Typhimurium but not the chlortetracycline-resistant *S.* Typhimurium.

## Data Availability

Data is privately held but can be made available upon reasonable request.
